# Supplementary material for: Deep convolutional and conditional neural networks for large-scale genomic data generation
Source: PLoS Comput Biol. 2023 Oct 30;19(10):e1011584. doi: 10.1371/journal.pcbi.1011584 (PMC10635570; doi:10.1371/journal.pcbi.1011584)

We thank the editor and reviewers for their time and valuable comments which helped us improve the manuscript. Please find below our detailed responses. Please note that lines we refer to in our responses below point to lines in the manuscript with red track changes. This is because LaTeX scripts alter line numbers slightly (a couple of lines) even though the composition is exactly the same between track and no-track versions except for the red color.

Reviewer 1:

- The authors fail to convince the reader that artificial genomes (AGs) without larger variations (indels, rearrangements etc) are interesting and for what applications such AGs are useful. Such genomes are quite far from real ones. A title indicating that it is about synthetic SNP profiles would seem more appropriate.

We thank the reviewer for their comments. We agree with the reviewer that all types of genomic variation are important and we are aiming for generating full artificial genomes with complete characteristics in the long run. However, our methods presented in this work include important steps for extending the generation process to large-scale sequences along with improvements in almost all statistics compared to previous models. In addition, since the largest portion of human genomic variation is based on SNPs, SNP profiles are widely utilized both in evolutionary and functional genomics for various types of research such as demographic analyses, natural selection scans and GWAS (1,2). We opt to keep the title since we call the outputs artificial genomes, both in the previous and current study, and we see each step as improvements toward a more complete generation. In addition, some other research has already adopted this terminology (3).

1. Collins, Francis S., Lisa D. Brooks, and Aravinda Chakravarti. "A DNA polymorphism discovery resource for research on human genetic variation." *Genome research* 8.12 (1998): 1229-1231.

2. 1000 Genomes Project Consortium. "A map of human genome variation from population scale sequencing." Nature 467.7319 (2010): 1061.

3. Dang, Meihua, et al. "Tractable and expressive generative models of genetic variation data." *bioRxiv* (2023): 2023-05.

- It is fairly straight-forward to simulate AGs with more conventional methods. The authors fail to explain why deep learning is needed. They should also compare to conventional algorithms. Here are some references:
X. Yuan, et al "An overview of population genetic data simulation", J. Comput. Biol., vol. 19, pp. 42-54, 2012.
A. Carvajal-Rodríguez, "Simulation of genomes: A review", Current Genomics, vol. 9, 2008. Show in Context CrossRef Google Scholar
C. J. Hoggart et al., "Sequence-level population simulations over large genomic regions", Genetics, vol. 177, pp. 1725-1731, 2007.
Chun Li, Mingyao Li, "GWAsimulator: a rapid whole-genome simulation program", Bioinformatics, Volm24, pp 140–142, 2008

In our previous work (1), we compared our models to conventional simulations and reported the main differences. Coalescent simulations, as well as most forward simulations, generate data which exist in a different space so cannot be used along with real data (there is no correspondence between real and generated variants). In addition, they are model-based so they require a priori assumptions and knowledge about the demographic history of the populations in question. To have a perfect match, these simulations would also require

integrating information regarding processing steps applied to real data, and knowledge of uncertainty at different levels (from sequencing pipelines to post processing pipelines). Some resampling based approaches can be used to generate good quality simulated data which can be used along with real data but they leak privacy significantly (1). Therefore, we would like to emphasize here that AGs we propose serve a completely different purpose from these simulations as we explained in the introduction: In essence, AGs (with future improvements) will hopefully be used as surrogate realistic alternatives to real databases without requiring direct access and without breaching the privacy of the donors. Now we include these aspects in the discussion part as well (L430-434).

1. Yelmen, Burak, et al. "Creating artificial human genomes using generative neural networks." *PLoS genetics* 17.2 (2021): e1009303.

- The paper assumes deep familiarity with the earlier work in reference 20 to understand the results section. The data and models are not properly introduced and the reader needs to consult the methods section first to make any sense of it.

We agree that the methods section is required to fully understand the work especially for readers who are not familiar with generative models so we moved the methods section before the results. We think that this reordering improves the reader experience and clarity of the manuscript.

- It is argued that this work is important for reasons of GDPR, but there is no discussion of privacy preservation. How can we be certain that the original genomes are not identifiable if one has access to the model or a large number og AGs? It is hardly enough to say that "None of the models have produced identical sequences and no full sequences were copied from the training dataset" (line 59).

We agree that providing guarantees for complete privacy preservation is very difficult. However, we had checked multiple criteria for privacy and overfitting: namely the AA_{TS} and the distribution of haplotypic pairwise differences. Figure 5d and figures S5, S6, S12 (previous manuscript figures S3, S4, S9 and S10) show these analyses. The sentence in line 59 (previous manuscript) was only referring to a single aspect in previous Figure S3 (now Figure S5) but we now extended this sentence to explain it in more detail (L272-275). In addition, we performed membership inference attacks along with privacy score analysis and now present these results in the manuscript (L321-337). These attacks demonstrate that in black-box case (where the attacker does not have access to the model weights) for WGAN, it is not trivial to identify samples in the training dataset even when the complete architecture is known to the attacker (L414-427).

Reviewer #2: The paper presented an updated and extensive analysis of a previous detailed exploration regarding generative models for creating artificial genomes preserving real biological properties and structures. The article is clear, well-written, and thorough in its analysis. The paper certainly deserves a high consideration for publication. I would like to provide some minor considerations and comments that may help enhance its comprehensiveness and readability.

Here are a few points:

1. In their previous work, the authors calculated a measure of privacy loss introduced by Yale et al. https://inria.hal.science/hal-02160496/.
It would be interesting to include also in this paper a comparison table between the old and new models regarding privacy loss. It would be valuable to understand how the new models perform in terms of privacy score.

We thank the reviewer for the comments.
First, it would not be feasible to directly compare all models on the 65K SNP dataset, as it is impossible to generate such large scale AGs with previous models due to computational limitations. What we could do instead is to compare the previous models for the 10,000SNPs with the new ones in terms of AA_{TS}, which can give an idea about possible privacy leaks. For the RBM, the scores are very similar with the previous models and for WGAN, AA_{TS} is increased which suggests more underfitting and therefore, is reassuring in terms of privacy.

Second, we are now including the privacy loss score (L327-330, Supp fig S14d). This score requires splitting the data into a train and a test set. Although we could do this easily in our previous work for the Estonian dataset, this study only exploits the 1000 Genomes data which has significantly less samples and significantly more variation, in particular for the 65K SNPs. This led us to evaluate the privacy loss of the new WGAN and RBM models using half of the 10,000-SNP data.

We additionally performed membership inference attacks on these same models and present these results in the manuscript (L321-337). Briefly, we observed possible privacy leakage for the RBM AGs but not for the WGAN AGs. Furthermore, black-box attacks on WGAN AGs indicate that, even if adversaries have access to the architecture and possess a mix of new and training samples, it is not trivial to detect which of the samples were in the training set.

2. Generally, I would recommend reorganizing the results of the various models, along with their respective scores, like the AA score, in a table format. Alternatively, consider placing figure S10 in the results section. This would make it easier for readers to grasp the information immediately.

We merged the previous S10 figure with AA score into the main figure (now Figure 5).

3. It would be beneficial to explicitly label the PCA densities in Figure 1a and 3a within the figures themselves, in addition to mentioning them in the figure legends. Similarly, for Figure 3c, including the label for LD within the figure itself would be helpful.

We now include headlines for the plots in the mentioned figures (now Figure 3 and Figure 5).

4. In the discussion section the authors better investigate the phenomenon of overfitting, specifically addressing the anomaly regarding the large difference between two terms of the AATS score (AAtruth and AAsyn). I propose relocating Table 1 to the results section, considering it presents new findings and it describe a new anomaly. Additionally, the analysis of the nearest neighbour chain should be presented in the materials and methods section.

We now present the nearest neighbour analysis results in the results section (L315-319) along with Table 1. We also added the methodology in materials and methods (L222-229).

5. In the methods section, the authors mention that during the training of the WCGAN, the train is stopped through a visual inspection of the overlap in PC space. While this method has its clear rationale, it seems quite arbitrary. I understand that it is beyond the scope of this paper to analyse potential alternatives in detail, but it would be interesting to discuss in the conclusion section any alternatives the authors consider feasible.

We now discuss the issue of stopping criteria in the discussion section (L371-383).

Reviewer #3: These authors present a novel approach based on Weierstrass GANs and convolutional processing for generating artificial genomes. They're building on their earlier

work, while integrating several new ideas. Considerable effort is made to try to characterize the new networks, and to what extent they're realistic.

I am generally positive to the work, but I have noted some issues in presentation that I would like to see resolved:

1. The spans of the datasets are specified in terms of the number of SNPs. Some discussion is made of their distribution of minor allele frequencies, but very little with regards to their location (although their extraction from the Omni 2.5 panel is stated). It would make sense to state the total span in terms of physical or mapping distans along chromosome 1. From a population structure perspective, the actual length, rather than the number of variants, should be crucial in terms of the amount of underlying variation found in the genomes.

We thank the reviewer for their comments. We now include the physical distances in the Materials and Methods section (10,000-SNP data chr15:27,379,578-29,625,035 - 2 Mbp and 65,535-SNP data chr1:34,247-81,813,279 - 80 Mbp) (L47-49).

2. Color-coded scatter plots are used extensively in the main text and supplementary figures, with the exception of a few isobar plots. For example, Figure 1 a is using isobars, while 1 b is using scatter markers. With the very light transparency and high density in the center of these plots, it is very hard to even qualitatively judge whether the median, or mean, of the SNP distribution actually tracks the supposed center line or not. It should also be clarified whether the correlation metric stated is computed against the theoretical line, or a fitted version. After all, any linear correlation can end up with a high correlation coefficient, while we want to know if we reproduce y=x, not any y=kx+b. The presence of "label=" in some captions are distracting.

We updated previous Figure 1b and and 3b for better visualisation (now Figure 3b and 5b). The correlation metric we used was Pearson's r and we now include this information along with linear regression slope and intercept in the figure captions.

3. Along the same lines, in plots where multiple datasets are shown within the same plot, such as in Figure S11, it's problematic to not shuffle the order of the markers. By shuffling the z order in the center and rightmost panels in that plot, it would be more immediately clear to what extent the various distributions coincide. This applies to several other figures as well, including S7.

Shuffling the z order in general generates visually confusing plots so instead we adjusted the size and opacity of the points for previous Figure S11 (now Figure S13) which now shows more clearly where the distributions overlap. It is also important to note that for these plots a single PCA was performed on the combined real/generated data. The left panel shows a subset of the points, i.e. the positions of the real genomes only, to ease direct comparisons. In other words, the gray points are identical across the 3 plots (left, middle, right).

For previous Figure S7 (now Figure S9), we now instead provide separate figures for CRBM and WGAN. For previous Figure S2 (now Figure S3), it is unfortunately not trivial to make either of these adjustments since there are 5 different categories. However, the presented 3-point correlation values reflect well how the generated data matches this characteristic of the real data.

4. I think that there is reason to be cautious about the privacy guarantees, both from sets of generated artifical genomes, and especially if the underlying generating model would be distributed. Backpropagating attacks based on short haplotype excerpts, for example, could indicate whether a biological relative of an individual was memoized from the training set and reproduce larger parts of its genome, at least hypithetically. I find that the final paragraph in the discussion acknowledges this point to some extent, while the abstract, on the other hand, makes bolder claims regarding privacy.

We agree that it is difficult to provide privacy guarantees and we updated our statement in the abstract. Furthermore, we performed membership inference attacks and now include more insights into privacy leakage (L230-247, L321-337, L414-427). Briefly, we demonstrate that if model weights are not shared, it would be difficult for adversaries to detect genomes in the training data for the WGAN model. However, we understand that this is only one type of attack and other possibilities should be explored extensively before AGs can be employed in real life cases.

5. While I understand the challenges involved, I find it unfortunate that no ablation study is performed. We rather see a discrete step between the old GAN approach and the new WGAN approach, while in fact there are multiple, distinct contributions that have been included.

We agree with the reviewer that an ablation study would be insightful but the models were developed with hundreds of different architectural and hyperparameter combinations, starting initially from shallower networks with smaller input sequence lengths. In addition, it is very difficult to make meaningful comparisons between different models if both models do not work. As an example, below PCA figures result from the training of two earlier models.The only difference between these models was the packing method (1) for reducing mode collapse (Figure 1 without packing, Figure 2 with packing) and neither of these models generated plausible haplotypes (based on PCA assessment) even after thousands of epochs of training. We opted to keep the packing for the further trials as it seemed to improve mode collapse (the generated blue points are more widely spread in Figure 2) but it is not trivial to assess the effect of this method without extensive analyses, especially considering very long training times (5-7 days for the 65,535 SNP data). Furthermore, we relied on literature for justification of certain updates such as the Wasserstein loss as it has been suggested to provide better training stability (2). However, it is an interesting lead for a future comparative study to see which techniques and architectures are suitable for generative modelling with genomic data.

Figure 1.

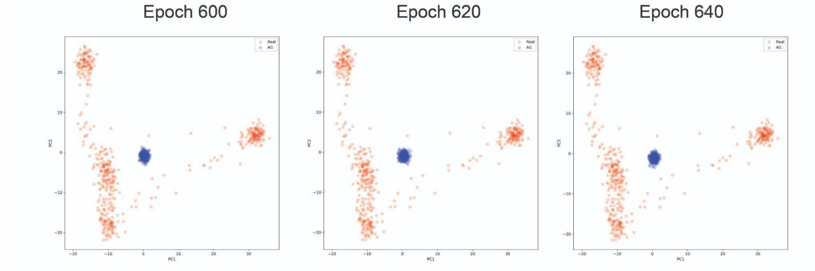

Figure 2.

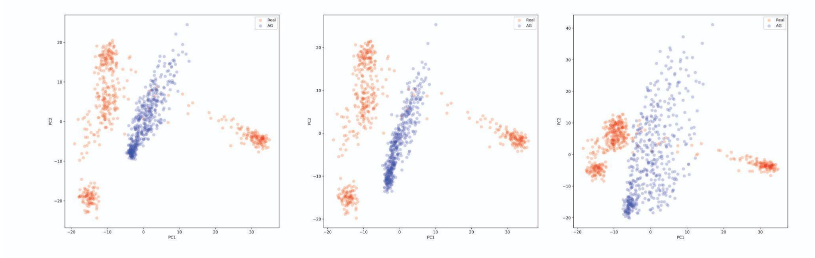

1. Lin, Zinan, et al. "Pacgan: The power of two samples in generative adversarial networks." Advances in neural information processing systems 31 (2018).

2. Arjovsky, Martin, Soumith Chintala, and Léon Bottou. "Wasserstein generative adversarial networks." International conference on machine learning. PMLR, 2017.

6. What constitutes a good artificial genome can of course be hard to quantify. I find the LD plot S1 to be both convincing and concerning in this regard. Other possible metrics would be the reproduction of specific short k-mers (so not only maintaining pairwise LD, but identical specific short-range haplotypes). Since the WGAN critic has been trained, it could also be illuminating to at least verify that the critic fares worse at discriminating between WGAN AGs and real genomes, relative to VAE AGs vs. real genomes... or is the critic so inherently wed to the WGAN that it is blind to the drawbacks of the other methods?

We had computed 3-point correlations (previous Figure S2 and S7, now S3 and S9) and observed that they were well reproduced in the generated genomes which, when the 3 SNPs are successive or very close by, gives an insight on short-range haplotype preservation. We now also check the correlation between real and artificial 4-mer and 8-mer motifs and include these results in the manuscript (L267-271, L303-304; Figure S4 and S10). As suggested by the reviewer, we also assessed the WGAN critic against AGs generated by other models. The below figure is the distribution of the critic score for these AGs and real genomes. It was interesting to see that although the distribution of the score is clearly different between real and generated datasets, the separation is not absolute in many cases. Especially for AGs generated by the previous RBM model (RBM_prev), we see that the critic does not perform well (i.e., not able to accurately distinguish between fake and real). We may infer from this that the critic might not be reliable for distinguishing all types of real/fake data points and the generated data should be assessed by other metrics. As the reviewer hypothesized, the critic might be partially blind to the drawbacks of datasets generated by other models. We opt to not include this result in the manuscript as it can be confusing to the reader.

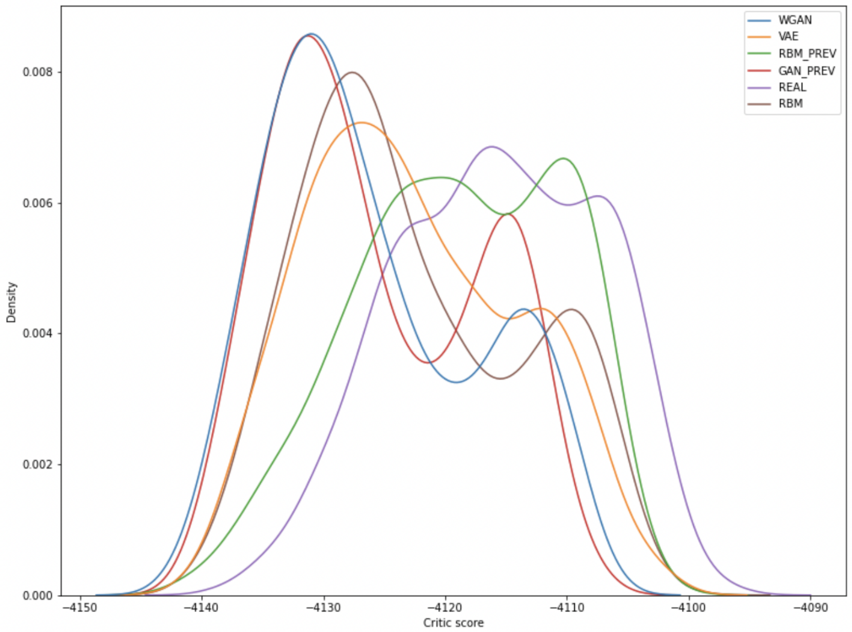

Supplement: Attachment — Submitted filename: response_to_reviews_yelmen_deep_2023.pdf [file pcbi.1011584.s016.pdf]
