# Supplementary material for: Deep convolutional and conditional neural networks for large-scale genomic data generation
Source: PLoS Comput Biol. 2023 Oct 30;19(10):e1011584. doi: 10.1371/journal.pcbi.1011584 (PMC10635570; doi:10.1371/journal.pcbi.1011584)
Supplement: Attachment — Submitted filename: response_to_reviews2_yelmen_deep_2023.pdf [file pcbi.1011584.s017.pdf]

We thank the editor and reviewers for their valuable comments and help to improve the manuscript. Below are our responses.

Reviewer #2: I thank the authors for their effort in their additional analyses and for satisfactorily responding to all my comments, especially adding the privacy loss score and performing membership inference attacks. The current version of the manuscript is definitely more complete and clearer in structure and exposition.

We thank the reviewer for their comments.

Reviewer #3: I appreciate the authors edits and their comments (including the extra critic score data in the reviewer responses). In general, I am satisfied with this state. I also find it crucial to show the slopes, since a slight deviation from the main diagonal would be almost imperceptible, but indicate a serious problem in e.g. the allele frequency plots.

My remaining thoughts are mainly also relating to figures and the presentation of data.

In Figure 3B, the y scale is different for the various plots in the lower set of subpanels in subfigure b. This is arguably due to outliers from some of the methods, but this artifact makes visual assessment of the data presented harder. When pairs of methods are compared, such as in Figure 5 b, I still think that it could make sense to join them in a single plot, IF shuffled z-ordering is implemented. I don't agree with the authors that this makes the result more visually confusing. If it is confusing, it's related to the nature of the overlap between the distributions. Such misleading covering of distributions is also clearly present in current supplementary Figure S3, where the RBM dominates the diagonal partially due to being drawn last. I think a Figure like S9 would also be more informative if the scatter point clouds were presented in a single figure.

This stackexchange thread gives a superficial illustration of the issue I'm referring to https://stats.stackexchange.com/questions/11984/how-can-i-remove-the-z-order-bias-of-a-coloured-scatter-plot (see especially the response from user bluenote10).

We thank the reviewer for their comments. We updated figures 3b, 5b and S14b so that the y-scale is fixed for all the lower panel plots. In addition, we updated figures S3 and S9 as the reviewer suggested using shuffled z-order. For Figure 5b, we tried the same approach but we opted to keep the original version (i) to preserve consistency with Figure 3b and (ii) because we believe the original version is more informative in terms of clearer visualisation of variance and density for separate classes. Due to the large number of points, these aspects are relatively less clear in the z-order shuffled scatter plots presenting both classes. Nevertheless, we below share the plot with shuffled z-order for Figure 5b. As an example, in the left figure, there is a visual bias which wrongly suggests a lack of red points on the diagonal compared to the periphery (which is not the case if you check Fig. 5b) and in the right figure, it is not easy to assess the density for the lowest frequency alleles relative to the highest frequencies for a given dataset due to the overlap. We hope these points clarify our decision.

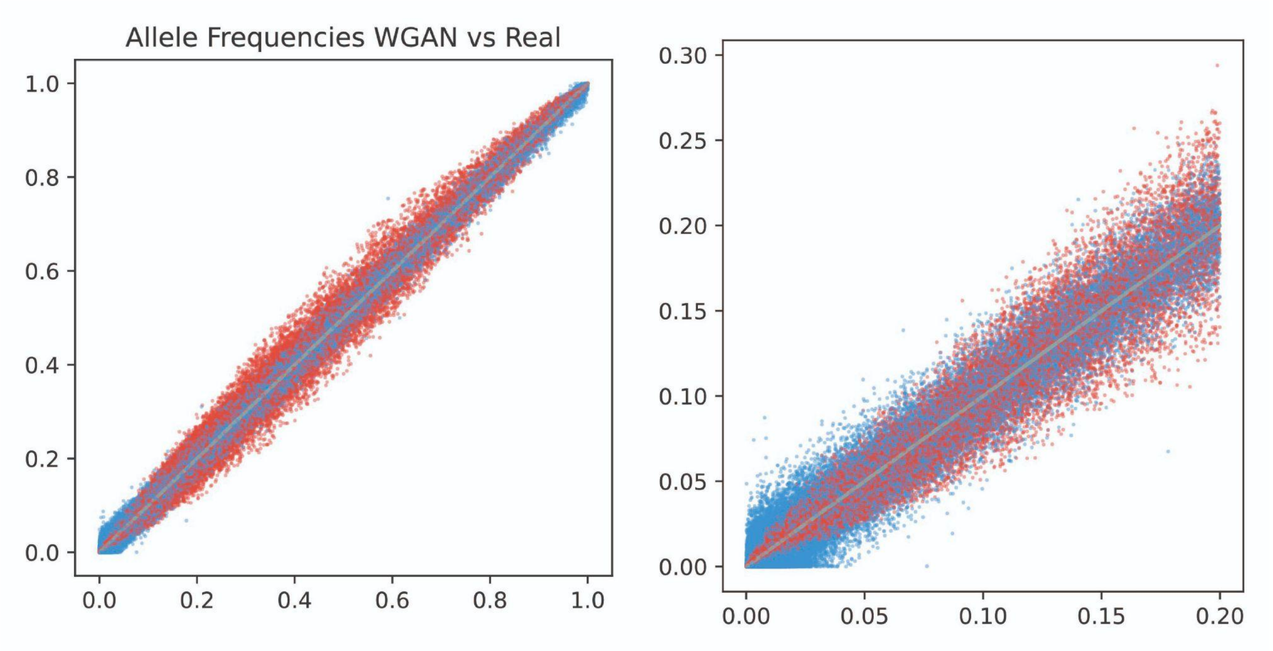
Allele Frequencies WGAN vs Real
0.0
0.2
0.4
0.6
0.8
1.0
0.00
0.05
0.10
0.15
0.20
0.25
0.30
